# Alzheimer's disease biological domain sub‐stratification enhances the precision of functional analyses

**DOI:** 10.1002/alz.71403

**Published:** 2026-06-10

**Authors:** Gregory A. Cary, Sai Sruthi Amirtha Ganesh, Laura Heath, Karina Leal, Martin Kampmann, Frank M. Longo, Robert R. Butler, Allan L. Levey, Gregory W. Carter, Jesse Wiley

**Affiliations:** ^1^ The Jackson Laboratory Bar Harbor Maine USA; ^2^ University of Kansas Lawrence Kansas USA; ^3^ Sage Bionetworks Seattle Washington USA; ^4^ University of California San Francisco San Francisco California USA; ^5^ Stanford University Medical School Stanford California USA; ^6^ Emory University Medical School Atlanta Georgia USA

**Keywords:** Alzheimer's disease, biodomain, Gene Ontology, proteomics, pseudotime, subdomain

## Abstract

**INTRODUCTION:**

The Target Enablement to Accelerate Therapy Development for AD (TREAT‐AD) bioinformatics pipeline employs a rank‐and‐organize strategy. Disease‐associated genes drive enrichment of large AD‐linked endophenotypes. However, these biological areas were too large to promote hypothesis development or target identification. Here we delineate subdomains that map to, and enrich, specific biological processes.

**METHODS:**

To refine the biodomains into more focused areas, we built *κ* networks out of the Gene Ontology terms in the biodomain and employ shared gene annotation between terms to determine edge weights. *κ*‐value filtration enabled us to identify data‐driven subdomains, which we employed in an analysis of TREAT‐AD harmonized datasets.

**RESULTS:**

The subdomain enrichment highlights core areas of biological impairment within the biodomain space and facilitates a deeper interpretation of large‐scale multiomic datasets.

**DISCUSSION:**

The subdomain mapping of AD‐risk‐associated processes may facilitate an open‐source, open‐science shareable resource for the comparison of large datasets for the formulation of future hypotheses and identification of therapeutic targets.

## Highlights

- Kappa‐network modeling defines new AD biological subdomains.
- Subdomains enhance interpretation of multiomic functional enrichment analyses.
- Subdomains demonstrate greater biological process resolution than biodomains.
- Disease‐informed subdomains bridge discovery and translational applications.
- Open‐source framework facilitates cross‐study AD data integration.

## 1 BACKGROUND

Research in recent decades has underscored the multifactorial nature of Alzheimer's disease (AD) across multiple biological systems. Large‐scale genomic, transcriptomic, and proteomic studies from National Institute on Aging (NIA)‐funded consortia – including the Accelerating Medicines Partnership for Alzheimer's Disease (AMP‐AD) – have generated vast data resources that provide an unprecedented opportunity to define the contributions of each of these biological systems to AD pathogenesis. However, biological interpretability – including effective integration across datasets and translating molecular associations into mechanistic insights – remains a major challenge. To address this challenge, the Target Enablement to Accelerate Therapy Development for AD (TREAT‐AD) consortium has developed the biological domains of AD.^1^

The original biological domains (biodomains) were developed to capture common molecular endophenotypes in a clear, intelligible, and objective manner. We identified core endophenotypes associated with disease and exhaustively defined these endophenotypes using Gene Ontology (GO) terms. This approach enables any genetic, transcriptomic, or proteomic dataset to be aligned to broad areas of AD pathology. Thus, this framework provides a unifying map linking system‐level data to the biological processes disrupted in AD. While the biodomains facilitated biological interpretation, the broad scope of many domains limited the ability to pinpoint specific processes relevant to mechanistic hypotheses or therapeutic targeting. To focus the biological precision of the methodology, we developed a new iteration of the AD biodomains by fragmenting each domain into smaller, more specific components – termed AD subdomains. Here, we define an AD subdomain as a functionally coherent cluster of GO terms within a parent biodomain, identified using kappa‐network fragmentation of shared gene annotations.

Multiple approaches for subdomain identification were explored, but we ultimately adopted a network‐based method that quantifies the degree to which disease‐associated genes are shared between GO terms. This allowed us to build network representations of each biodomain in which GO terms are connected by weighted edges reflecting shared AD‐enriched genes. Selective removal of weaker edges fragments each network into clusters of strongly associated terms, each representing a coherent biological subprocess. These networks were constructed two ways to ensure consistency of the identified subdomains: first, starting with the enriched GO terms identified in the gene set enrichment analyses (GSEA); and secondly, we developed *κ* networks starting with the leading edge genes. In both models the GO terms are the nodes, and the degree of shared gene annotation between GO terms is the *κ* value defining the edge weight. Using this approach, we refined the original 19 biological domains to 110 subdomains, ultimately increasing biological resolution and reducing redundancy.

Although the subdomain framework builds on the TREAT‐AD biodomains and target risk score (TRS) analyses described previously,^1^ the present study provides several distinct advances. First, we introduce a kappa‐network fragmentation strategy to systematically resolve broad biodomains into finer‐grained biological subprocesses. Second, we provide a curated subdomain resource that reduces GO term redundancy while enabling higher‐resolution subprocess‐level interpretation. Third, we demonstrate that subdomain‐level modeling improves interpretability across multiple independent datasets and modalities. Together, these advances extend the original biodomain framework toward higher‐resolution molecular fingerprinting and improved prioritization of mechanistically relevant processes in AD.

**RESEARCH IN CONTEXT**

1. **Systematic review**: The TREAT‐AD bioinformatics pipeline utilizes a rank‐and‐organize approach to nominate AD targets for investigation. We employ multiomic scoring for AD risk to rank genes and an alignment to AD‐associated endophenotypes to organize them into discrete biological areas. The need for increased resolution led to the data‐driven enhancement reported here, mapping datasets to more refined and actionable biological processes called AD subdomains.
2. **Interpretation**: The data‐driven identification of the AD subdomains allows a more refined and accurate interpretation of large datasets and enables a more meaningful comparison between datasets. This advancement leverages increases in specificity and sensitivity to identify more actionable areas of enrichment, promoting more informed target identification and hypothesis formulation.
3. **Future directions**: Constantly emerging and expanding new techniques require advanced methods to perform multiomic alignment to a common conceptual framework. We will be further developing and testing the AD subdomains for this purpose.

We employed the new subdomains within the TREAT‐AD analytic pipeline to characterize enrichment across large human datasets. We apply the subdomains to characterize enrichment profiles using the AD TRS framework described previously,^1^ extending those analyses from broad biodomain‐level interpretation to higher‐resolution subprocess mapping. A major challenge in AD research is the reliance on *post mortem* tissue to reconstruct the *ante mortem* disease pathology. We applied the biodomains and subdomains to characterize previously reported disease pseudo‐progression trajectories in AMP‐AD cohorts.^2^ Finally, we show the utility of the subdomains for interrogating functional perturbation data, a critical step in identifying candidate therapeutic targets. In each case, subdomain‐level characterization was able to resolve and refine enrichment profiles observed at the biodomain level, resolve directional patterns of dysregulation, and pinpoint processes showing potential restoration or reversal. Together, these analyses demonstrate that subdomain‐level modeling increases interpretive precision, enhances alignment across datasets, and provides a scalable framework for mechanism‐driven therapeutic discovery in AD.

## 2 METHODS

### 2.1 Overview and rationale

The goal of the subdomain project is to create interpretable, disease‐informed endophenotypic traits associated with AD. These traits can then be used for high‐resolution characterization and cross‐dataset multiomic alignment. Specifically, our objective was to enumerate subprocesses within each biological domain to achieve a more precise depiction of the biology altered across disease trajectory. Because the original construction of biological domains relied heavily on GO term‐based annotations, we initially explored standard ontological subsetting approaches. However, simple partitioning of the ontology proved impractical: The biological domain concepts map across disparate areas of the ontology, and the complex parent–child relationships in GO hindered systematic refinement. We therefore adopted a network‐based approach, leveraging Cohen's kappa analysis of shared gene annotation between GO terms to build domain‐specific GO term networks. These networks were subsequently filtered into subentities representing distinct biological contributors to disease pathology and progression.

#### 2.1.1 Top‐down construction of biological domain‐specific kappa networks

For each biological domain, we constructed networks from the constituent GO terms and enriched genes identified in the TREAT‐AD case‐control multiomic risk‐informed gene prioritization framework, termed the TRS.^1^ If an enriched gene was annotated to multiple GO terms within a biological domain, this annotation created an edge linking those two terms. The resulting domain‐specific networks were therefore defined by both GO terms and enriched gene annotations. To define the networks, we used GSEA‐derived leading‐edge genes (those driving enrichment maxima) for GO terms with false discovery rate (FDR)‐adjusted *p* values ≤ 0.05. Edge weights were assigned based on Cohen's kappa score, which quantifies agreement between GO terms:

$$\kappa =\left(p_{o}-p_{e}\right)/\left(1-p_{e}\right),$$

where p_o_ is the observed agreement and p_e_ is the expected agreement by chance. Although Cohen's kappa was originally designed to assess rater agreement, it has been widely used for constructing gene‐linked GO term networks, such as we employ here.^3^, ^4^ The outcome was a data‐driven network of GO terms for each biological domain, designed to minimize curator bias and highlight subprocesses most implicated in AD biology.

Edge weights reflect the strength of the relationship between GO terms, with higher kappa scores indicating a greater proportion of shared genes. To resolve networks into meaningful clusters, we removed weaker edges with a threshold kappa score ≤ 0.5, which effectively fragmented the biological domain term networks into biologically coherent clusters. To assess the robustness of this procedure, we evaluated fragmentation across a range of *κ* thresholds, confirming that major subnetwork structure emerges consistently with increasing stringency (Figure S1B). Clusters containing no more than four GO terms were excluded from consideration to avoid spurious small modules. In some cases, term fragmentation resulted in clusters that were conceptually related but lacked uniformly strong edges. When (1) the biological relationship was clear to an expert evaluator and (2) lowering the threshold restored relevant connections, we merged the related terms into one subprocess. For some smaller networks (e.g., APP Metabolism, Tau Homeostasis), weaker thresholds (0.3 < *κ* < 0.5) were sometimes required to achieve informative fragmentation. The rationale for using a bounded range of *κ* thresholds derives from the basic properties of GO annotation networks. Gene‐to‐GO‐term relationships are bipartite, and projecting genes onto GO terms induces dense connectivity because pleiotropic genes link multiple terms simultaneously. As biodomain size and annotation density increase, the distribution of *κ* edge weights shifts upward and hub density increases, making larger networks more resistant to fragmentation under a single fixed cutoff. Conversely, smaller networks fragment more readily. Because *κ* distributions vary systematically with network size and density, a universal *κ* cutoff would bias fragmentation outcomes. We therefore scaled *κ* thresholds empirically based on network coherence and density, while constraining all analyses to a consistent bounded range (0.3 ≤ *κ* ≤ 0.5).

#### 2.1.2 Bottom‐up network construction

As an independent check, we constructed bottom‐up *κ* networks using the same AD‐associated, TRS‐prioritized gene sets. Specific up‐ and downregulated gene sets were submitted to ClueGO, a Cytoscape‐based network‐modeling application.^3^, ^4^ Unlike the top‐down approach, this method was not constrained by the GO terms that were originally assigned to each domain and consequently generated broader enrichment patterns. Our goal was not perfect concordance between the two methods, but rather to assess whether the bottom‐up subnetworks recapitulated clusters similar to those observed in the top‐down approach. While we ultimately favor the constrained top‐down approach, the bottom‐up approach provided independent validation that identified gene set clusters highlight the same underlying biological subprocesses.

Bottom‐up networks tended to be larger and more diffuse. To increase interpretability, we employed several filters. First, we required ≥10% gene overlap and a *κ* value ≥ 0.4 for edges. Second, we restricted terms to those with ≥60% specificity for up‐ or downregulation (based on transcriptomic data, which had broader coverage than proteomic data). Third, we employed Resnik scoring within the SemSim software package^5^, ^6^ to collapse highly similar terms, while retaining GO terms that captured distinct subprocesses, to facilitate ranking of the term status relative to the GO within each submodule identified.

### 2.2 Subdomain harmonization

Harmonization between top‐down and bottom‐up frameworks was based on conceptual alignment, not strict numeric identity. Thus, inclusion of a conceptually similar subnetwork in both approaches was considered adequate. To avoid dependence on a single AD dataset, we combined the harmonized top‐down and bottom‐up results and then modestly expanded the GO terms defining each subdomain. Expansion was constrained to closely related processes within the same subject area. For example, if the Mitochondrial Metabolism subdomain centered on the tricarboxylic acid cycle, we included related processes such as succinate dehydrogenase activity, even if they did not appear in the AD‐enriched gene set. This procedure ensured that the subdomains were disease‐informed but not disease‐defined: anchored in AD data, but generalizable for future studies and applicable across related fields (e.g., psychiatry, where overlapping biology is common^7^, ^8^).

Subdomain harmonization was performed by independently fragmenting term‐centric and gene‐centric kappa networks and comparing the resulting clusters within each biodomain. Representative lead terms were selected based on (1) biological interpretability and conceptual coverage of the cluster, (2) GO hierarchy (i.e., selection of the most inclusive term capturing the cluster), (3) internal coherence of the cluster as reflected by shared gene membership and Resnik semantic similarity, and (4) relative TRS signal among constituent terms. Clusters were considered harmonized when they converged on the same biological process within the same parent domain. This approach was used as an internal reproducibility check across independent network constructions rather than to enforce strict mathematical equivalence. Across biodomains, the majority of nominated subdomains were supported by both approaches, with disagreements largely restricted to small or weakly connected clusters.

In total, this process refined the 19 parent biological domains into 110 subdomains (Table S1), while simultaneously reducing the overall number of GO terms by nearly 50%. This dual effect of increasing resolution while reducing redundancy provides a more precise and tractable tool for biological interpretation and therapeutic target prioritization.

### 2.3 Gene set enrichment analysis

We performed GSEA using the AD risk‐informed gene prioritization scores developed.^1^ Specifically, we used the TRS and component genetic and multiomic scores to rank gene symbols for GSEA and used the gseGO function from the clusterProfiler R package (version 4.10.1),^9^, ^10^ in positive score mode, against the org.Hs.eg.db (version 3.18.0). We also used the multiomic treatment effects from the omics modality‐specific meta‐analyses to perform GSEA for transcriptomics and proteomics using gseGO in standard score mode. Significantly enriched terms (adjusted *p* ≤ 0.05) were mapped to the biological domain definitions, including subdomains (syn25428992.10), using GO term accessions to link terms. We compared term enrichments between score modalities (i.e., genetics vs multiomics scores) or omic modalities (i.e., transcriptomic vs proteomic) for terms that were significant for both comparisons.

### 2.4 Disease pseudotime analyses

To establish biodomain and subdomain enrichments across AD pseudotemporal progression, we employed transcriptomic data from female donors in the ROSMAP and Mayo AMP‐AD cohorts from the original study by Mukherjee et al. Manifold learning was used to learn a trajectory through the samples in each cohort, and each sample was assigned to a separate branch (or state) of the trajectory. One‐way ANOVA with Tukey's Honestly Significant Difference was used to assess differential expression for all states relative to the reference state, with the largest proportion of healthy control samples. We used the output from these differential expression analyses to perform GSEA. The ANOVA effect size for all genes significantly differentially represented by samples at each state (ANOVA *p* ≤ 0.05), relative to state 1, was used to rank gene symbols. We used the gseGO function from the clusterProfiler package against the org.Hs.eg.db annotation database. Significantly enriched (adjusted *p* ≤ 0.05) GO terms were mapped to the biological domain definitions, including subdomains (syn25428992.10), using GO term accessions to link terms, and plotted biodomains that were significant across multiple states.

### 2.5 CROP‐seq data

To assess gene expression differences in response to gene perturbation experiments, we referred to the CRISPRbrain.org resource.^11^ Through the CRISPRbrain Application Programming Interface we downloaded gene expression data for all targets tested in Clustered Regularly Interspaced Short Palindromic Repeats‐activating (CRISPRa) and CRISPR inhibitory (CRISPRi) screens in neurons and microglia derived from healthy donor induced pluripotent stem cell (iPSC) lines. We defined a custom set of pathways for each biodomain and subdomain based on the genes annotated to each and then used the fgsea multilevel function from the fgsea R package (version 1.30.0)^12^ to test for enrichment for each unique target, CRISPR mode, and cell type combination. Significant enrichments (adjusted *p* ≤ 0.05) were plotted for each subdomain within the Synapse and Immune Response domains for perturbations that had at least six significant subdomains.

## 3 RESULTS

### 3.1 Generation of AD subdomains

**FIGURE 1:**
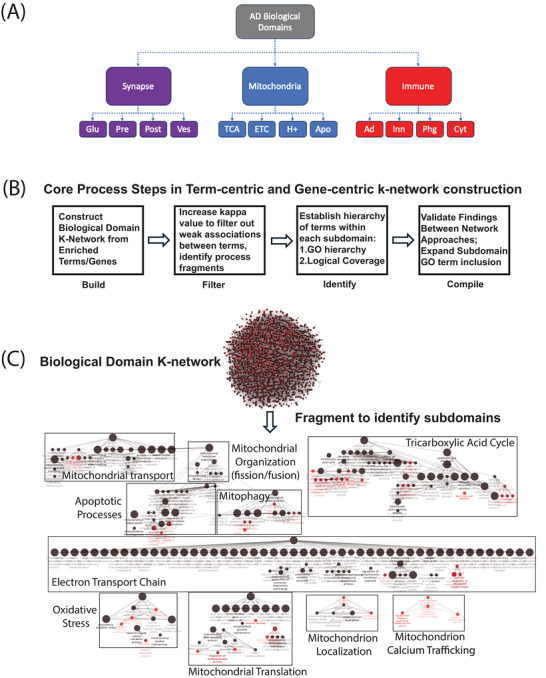
Development of Target Enablement to Accelerate Therapy Development for Alzheimer's Disease (TREAT‐AD) subdomains. We developed a process to leverage the disease state information to inform about which components within the Alzheimer's disease biodomains are contributing most to disease association. (A) Illustration of conceptual goal, where biological domain is broken down into more useful and intelligible discrete biological processes. (B) Diagram of workflow for development of two types of kappa networks, those built from the enriched Gene Ontology (GO) terms and those derived from the leading edge and *p*‐adjusted post‐false disvoery rate‐correction gene sets. (C) Example of process, in which mitochondrial metabolism kappa network is deconstructed into its constituent subdomains.

The original AD biodomains were designed to organize large datasets into coherent endophenotypic areas. However, their breadth has limited their utility for focused hypothesis testing or therapeutic development. To address this, we subdivided each biodomain into discrete, biologically interpretable subdomains directly linked to AD pathogenesis (Figure 1A). The overall workflow for this process is shown in Figure 1B and described in detail in the Methods section.

Because the GO structure contains multiple hierarchical levels and complex parent–child relationships that do not guarantee inheritance of biological function, simple ontological subsetting was not feasible. Instead, we employed a kappa‐network approach, in which Cohen's kappa (*κ*) coefficient – traditionally used to measure rater agreement – was adapted to quantify shared gene annotations between pairs of GO terms. In this framework, GO terms are nodes and the *κ* values representing gene annotation overlap serve as weighted edges. All 19 biodomains were modeled as network objects by applying Cohen's *κ* to quantify shared gene annotations between enriched GO terms. This generated dense, interconnected networks of GO terms within each domain.

Gradually increasing the stringency of *κ*‐filtering (typically removing edges with *κ* < 0.4 to 0.5, depending on network complexity) caused the networks to fragment into smaller, well‐defined subnetworks that formed the basis for the subdomain definitions. An example of the progressive filtration process is shown in Figure S1A–C. Fragmentation occurred progressively with increasing *κ* stringency, with network structure resolving into smaller clusters rather than collapsing abruptly, supporting the robustness of the thresholding procedure (Figure S1B). The fragmented subnetworks displayed strong internal functional coherence, consistent with high gene conservation between terms that determined their formation. Importantly, the genes linking terms were derived from TRS analyses, ensuring that disease‐state information guided the identification of biologically relevant subprocesses.

As the networks were filtered and decomposed into submodules, we next characterized the biological nature of each resulting subdomain. To assign each cluster a subdomain identity, we considered two complementary criteria: (1) ontological ascendency, selecting the GO term that most broadly captured the cluster's biological concept, and (2) logical coverage, ensuring the chosen term represented most constituent functions. In most cases, these criteria converged on the same representative term. Finally, we cross‐validated the resulting subdomains against those produced by a parallel, independent bottom‐up network‐development pipeline to confirm consistency and data‐driven reproducibility.

### 3.2 Validation with gene‐centric networks

As detailed in the Methods section, we also constructed gene‐centric (i.e., bottom‐up) networks to complement the term‐driven analysis. In this approach, the leading‐edge genes from each biodomain were used to build de novo networks based on the GO terms associated with those genes. Because individual genes often exhibit pleiotropic functions, some GO terms originated outside the biological domain; these were subsequently removed to maintain domain specificity. This bottom‐up strategy is inherently more sensitive to term nomination, as it is not restricted to the predefined set of enriched terms. Consequently, it serves as an independent, data‐driven validation on the term‐based approach, allowing us to identify potentially overlooked biological processes. An example of a gene‐centric network for mitochondrial metabolism is shown in Figure 1C.

### 3.3 Subdomain harmonization and characterization

**FIGURE 2:**
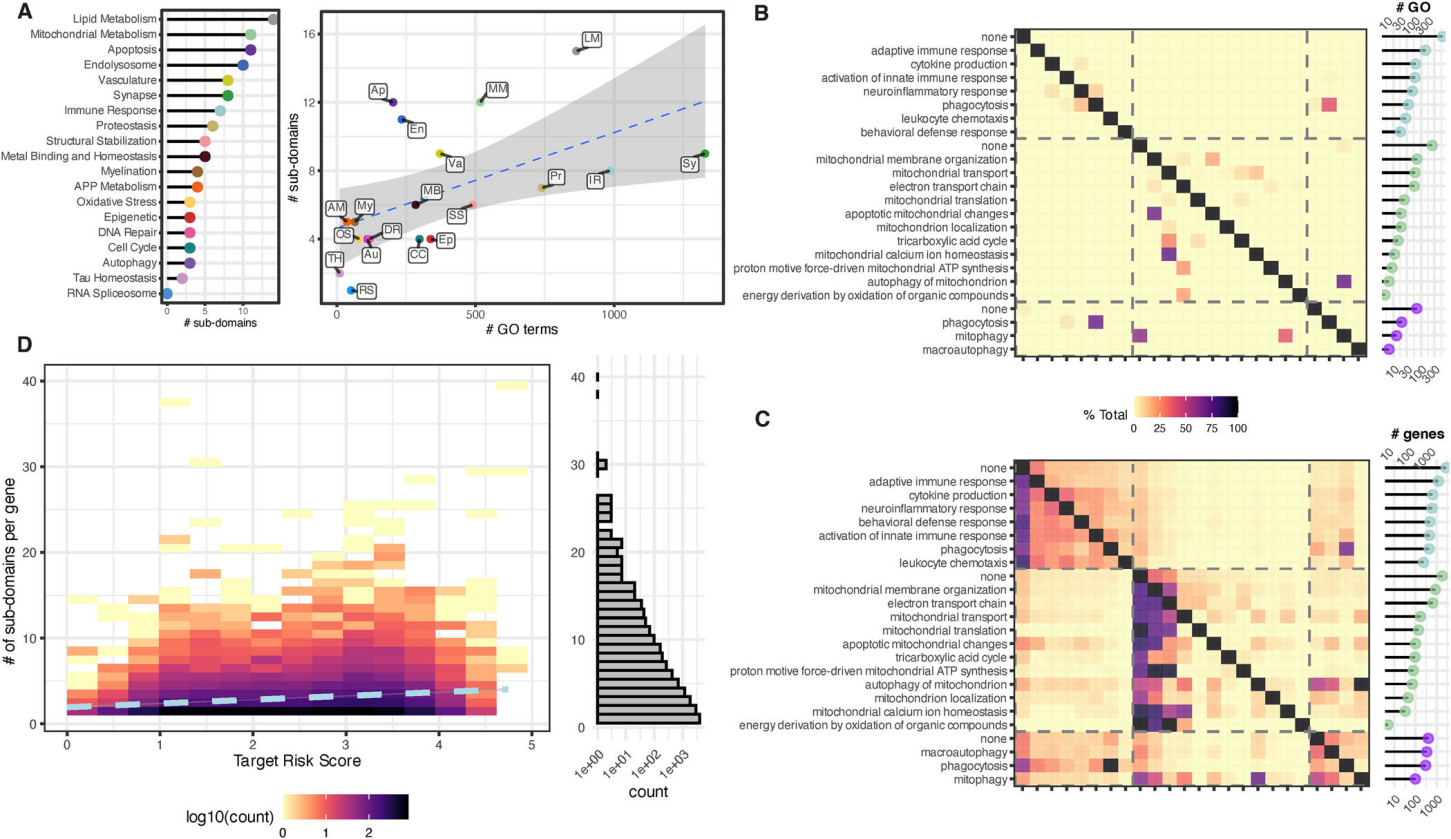
Demographics of Alzheimer's disease (AD) subdomains. (A) The fragmentation of all 19 AD biodomains resulted in 110 subdomains, with the number of subdomains per domain shown for each parent domain (left) and the correlation between the number of Gene Ontology (GO) terms in each parent biodomain and the number of derived subdomains (right, Pearson's *r* = 0.5, *p* = 0.016). Heatmaps showing overlap of GO terms (B) and genes (C) between subdomains from Immune Response, Mitochondrial Metabolism, and Autophagy domains. The lollipop plot on the right shows the number of GO terms or genes in each subdomain, and the fill color on the heatmap represents the percentage of the total GO terms or genes that overlap. The ordering of subdomains is the same on the *x*‐axis as on the *y*‐axis. (D) Many genes are annotated to multiple subdomains (histogram, right panel), and there is a weak but significant correlation between gene target risk scores and the number of subdomains to which genes are annotated (left panel, Pearson's *r* = 0.152, *p* = 4.7 × 10^−62^).

Both term‐driven and gene‐centric networks were constructed for all 19 biological domains, filtered by *κ* value, and examined to nominate AD subdomains. The two approaches produced highly consistent subdomain structures, demonstrating methodological robustness to network construction strategy. This robustness is illustrated for the Mitochondrial Metabolism biodomain (Figure S2). Harmonization between the two methods (Figure 1B, step 3) yielded 110 subdomains across the 19 AD biodomains (Figures 2A and S2A, Table S1). Harmonization between term‐centric and gene‐centric kappa‐network approaches demonstrated strong conceptual concordance across biodomains. Although the two methods do not produce identical cluster boundaries, they consistently recovered the same core biological subprocesses. The goal of harmonization was not to enforce strict equivalence between the approaches but to confirm that identified subdomains were reproducible across independent network constructions within the same biodomain.

Of the 6837 GO terms that originally annotated to the biodomains, 3267 (47.8%) were retained within at least one subdomain. This reflects the guiding principle of subdomain development – refining large, disease‐informed domains into smaller, more interpretable functional units. Overall, this process reduced by more than half the number of terms, while increasing biological stratification more than five‐fold (from 19 biodomains to 110 subdomains). The number of subdomains produced by each parent domain was proportional to its size (Figure 2A, second panel; Pearson's *r* = 0.5, *p* = 0.016).

GO term overlap between subdomains was generally sparse (Figure S3). Most overlap occurred among subdomains within the same parent domain, though limited cross‐domain overlaps were observed. For example, subdomains related to phagocytosis overlap between the Autophagy and Immune Response domains (14 of 35 and 20 terms, respectively), and mitophagy‐related subdomains overlap between the Autophagy and Mitochondrial Metabolism domains (five of 13 and seven terms, respectively) (Figure 2B). Importantly, none of these overlaps were complete, indicating nuanced contextual differences in term composition depending on the parent domain.

At the gene level, most TRS‐scored genes (52.2%) were not annotated to any subdomain. Among those that were, 67.3% mapped to more than one subdomain, reflecting pleiotropic gene functional annotation (Figures 2C and S4). The number of subdomains to which a gene was annotated showed a weak positive correlation with AD TRS (Pearson's *r* = 0.152, *p* = 4.7 × 10^−62^; Figure 2D), comparable to the correlation observed at the biological domain level (Pearson's *r* = 0.158, *p* = 2.8 × 10^−80^).^1^

Gene‐level overlap across subdomains mirrored GO‐term relationships: Overlap was greatest within parent domains but still evident across domains, with patterns similar to the GO term overlaps noted above (Figures 2C and S4). This reflects the pattern we observed with gene‐level versus GO‐term‐level overlaps of the parent domains and reflects the pleiotropy of individual genes with multiple functions that cross biodomains boundaries. Together, these results indicate that while subdomains capture distinct biological processes, they remain interconnected through shared molecular components, providing a hierarchical but continuous representation of AD biology.

### 3.4 Subdomains refine and contextualize the molecular architecture of AD‐associated biology

A primary motivation for subdividing the biological domains was to enable more precise contextualization of functional enrichment analyses. Here, we demonstrate that the subdomain framework provides finer resolution and clearer biological interpretation than the parent biodomains by analyzing the TREAT‐AD disease‐association gene metrics described previously.^1^ Importantly, core subdomain enrichments recurred across independent measures of disease association (genetic vs multiomic), molecular layers (transcriptomic vs proteomic), pseudotemporal modeling, and perturbation datasets, providing evidence of cross‐dataset reproducibility.

**FIGURE 3:**
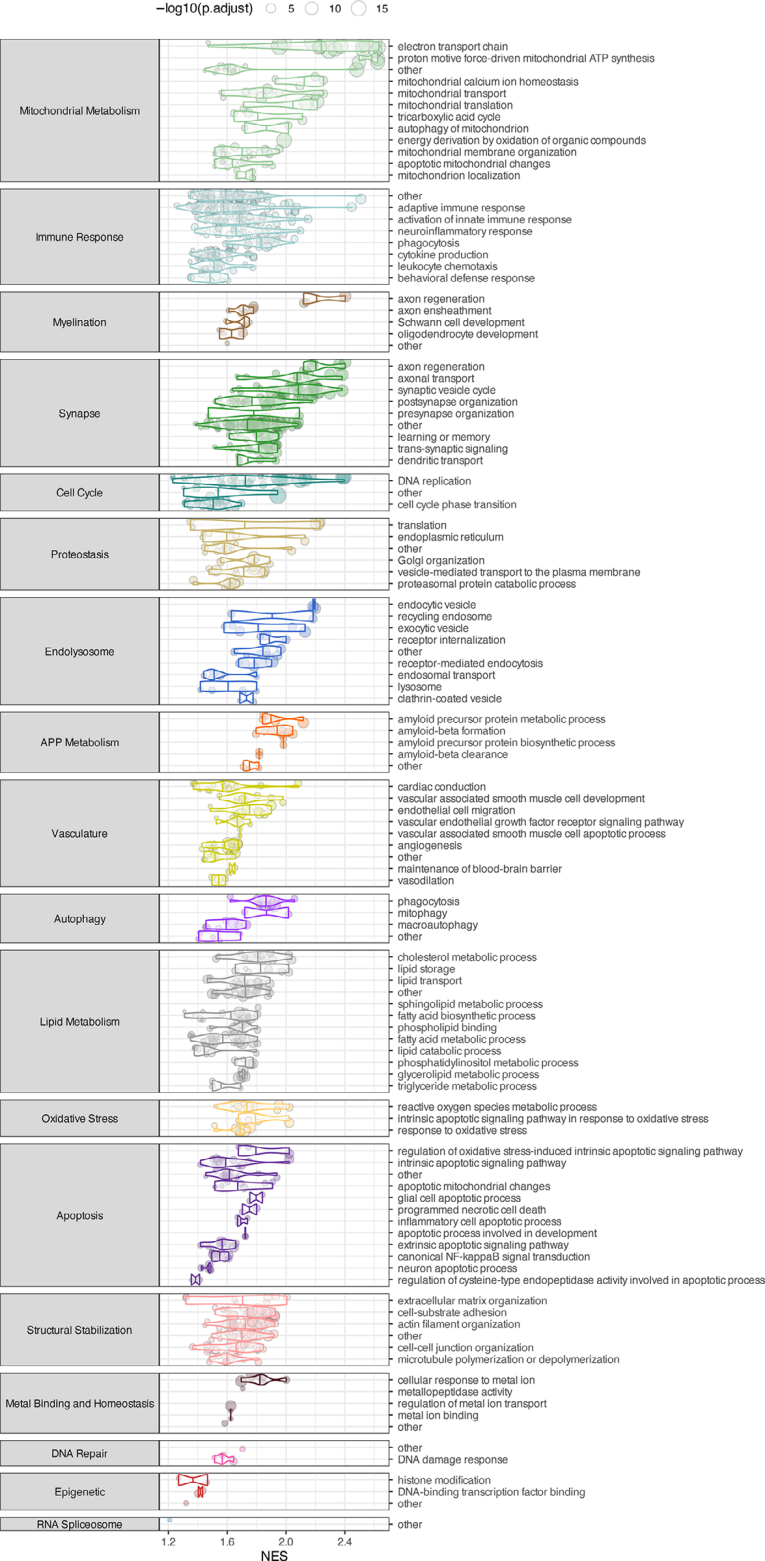
Target Enablement to Accelerate Therapy Development for Alzheimer's Disease (TREAT‐AD) target risk score (TRS) enrichment partitioned by subdomain. Statistics from gene set enrichment analysis using the TRS are shown for all AD biodomain Gene Ontology (GO) terms. Each point is a GO term from the indicated subdomain (right) within the parent biodomain (left facets), while the position along the *x*‐axis corresponds to the normalized enrichment score (NES), and the size of the point is scaled by the adjusted *p* value from GSEA, −log_10_ transformed. Biodomain terms not assigned to any subdomain are shown within the “other” category. Terms with larger NES values contain genes that are skewed toward the higher end of the TRS distribution. The subdomains are ordered on the *y*‐axis by the maximum NES identified from each subdomain.

We re‐evaluated the GSEA results using the various dimensions of AD association developed previously including the overall TRS (Table S2), as well as the genetic (Table S3) and multiomic (Table S4) component scores, along with the meta‐analyses of differential expression from transcriptomic and proteomic studies that are used to inform the multiomic score. Of the 2997 terms that are significantly enriched using the TRS to rank genes for GSEA, 1279 (42.7%) were captured within the biodomains, and 941 (73.6%) of these mapped to at least one subdomain (Figure 3). GSEA results mapped onto the subdomains reveal enriched biological patterns that are consistent with, but far more refined than, those seen with the parent domains (Figure 3). The biological domains with the largest number of significantly enriched terms were Immune Response, Synapse, and Lipid Metabolism.^1^ Correspondingly, the subdomains with the largest number of significantly enriched terms include *adaptive immune response*, *cytokine production*, and *activation of innate immune response* (Immune Response), *postsynapse organization* and *synaptic vesicle cycle* (Synapse), and *fatty acid metabolic process* (Lipid Metabolism). Subdomains with multiple significantly enriched terms and highest median normalized enrichment scores (NESs) include *electron transport chain*, *proton motive force‐driven mitochondrial ATP synthesis*, and *mitochondrial calcium ion homeostasis* (Mitochondrial Metabolism), *endocytic vesicle* (Endolysosome), and *synaptic vesicle cycle* and *axon regeneration* (Synapse).

Contrasting the enrichment results from similar analyses based on the Genetic (Table S3) and Multiomic (Table S4) association scores highlights subdomains that are differentially influenced by each modality (Figure S5). For example, terms from the *electron transport chain* subdomain (Mitochondrial Metabolism) are more significantly enriched using the multiomic score, terms from the *DNA replication* (Cell Cycle) subdomain are more significantly enriched using the genetics risk score, while terms from the *cell‐substrate adhesion* (Structural Stabilization) subdomain are equivalently enriched using both metrics. These risk‐informed modality‐specific enrichment patterns illustrate how the subdomain framework can disentangle overlapping molecular influences, revealing complementary layers of genetic susceptibility and disease‐state perturbation.

GSEA of the transcriptomic (Table S5) and proteomic (Table S6) datasets demonstrated clear and mostly consistent directionality, with positive NES value indicating upregulation and negative NES values indicating downregulation. Within the proteomic data, we observed strong bidirectional signals across the parent biodomains.^1^ The most pronounced downregulation occurred within the Mitochondrial Metabolism biodomain, particularly *mitochondrial translation* and *electron transport chain* subdomains. We also see profound downregulation of the Synapse biodomain that was further resolved using the subdomain framework. In particular, the *synaptic vesicle cycle* and *learning or memory* subdomains were the most downregulated.

**FIGURE 4:**
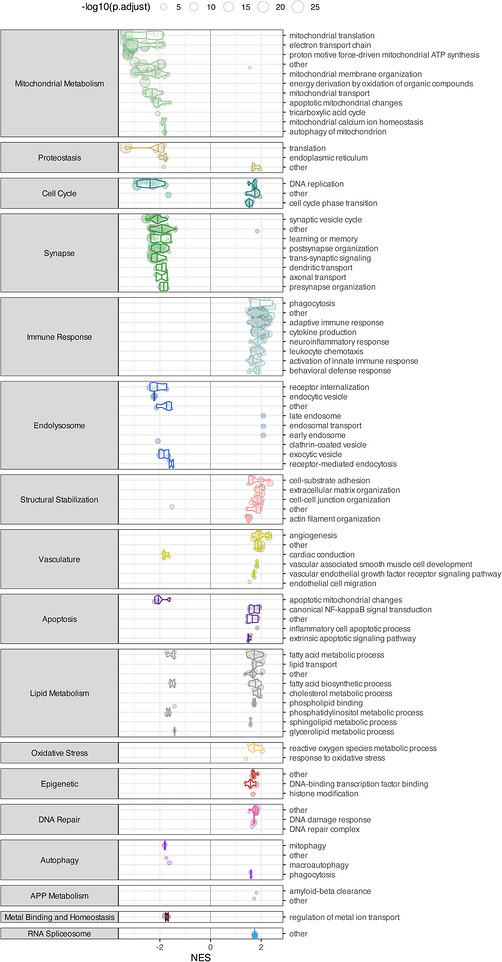
Proteomic meta‐analysis‐based enrichment partitioned by subdomain. Gene set enrichment analysis (GSEA) statistics for all biodomain Gene Ontology (GO) terms are based on proteomics meta‐analysis treatment effect. Each point is a GO term from the indicated subdomain (right) within the parent biodomain (left facets), while the position along the *x*‐axis corresponds to the normalized enrichment score (NES) and the size of the point is scaled by the adjusted *p* value from GSEA, −log_10_ transformed. Biodomain terms not assigned to any subdomain are shown within the “other” category. Term NES values indicate whether proteins from the term tend to be upregulated (positive NES) or downregulated (negative NES) in *post mortem* AD brains relative to controls.

Conversely, upregulated subdomains clustered predominantly into Immune Response, Structural Stabilization, and Lipid Metabolism (Figure 4). Within Immune Response, both *phagocytosis* and *adaptive immune response* showed the highest NES values. Structural Stabilization displayed elevated NESs across *extracellular matrix organization* and *cell‐substrate adhesion*, with relatively weaker upregulation of terms from the *actin filament organization* subdomain. This pattern suggests that structural remodeling in AD may primarily involve stabilization of extracellular interactions rather than through cytoskeletal rearrangements. The Lipid Metabolism domain is one that shows bidirectional enrichment at the biodomain level – some terms from the domain are upregulated, while others are downregulated. The subdomains help to distinguish the directionality of lipid‐related functions, with subdomains like *lipid transport*, *cholesterol metabolic process*, and *sphingolipid metabolic process* being uniformly upregulated and subdomains like *phosphatidylinositol metabolic process* and *glycerolipid metabolic process* being uniformly downregulated.

We can also contrast the directionality of enrichment between modalities to identify processes that are enriched in divergent directions based on transcriptomic or proteomic analyses. We found four subdomains with at least one divergent term (Figure S6). Terms from *receptor‐mediated endocytosis* and *receptor internalization* (Endolysosome) are downregulated in the proteomic dataset, but three of these terms – receptor‐mediated endocytosis, receptor internalization, and regulation of receptor mediated endocytosis – are upregulated in the transcriptomics datasets. Similarly, terms from the *fatty acid metabolic process* and *phospholipid binding* subdomains are upregulated in the transcriptomic datasets, while two of those terms (one shared by both subdomains) – phospholipid metabolic process and glycerophospholipid metabolic process – are downregulated in the proteomics datasets. These instances of divergent enrichment directionality likely represent points of molecular disequilibrium, where transcriptional activation is not matched by corresponding protein‐level changes, highlighting subdomain‐specific points of regulatory disruption in AD pathogenesis.

### 3.5 Pseudotemporal analysis of AMP‐AD cohorts

**FIGURE 5:**
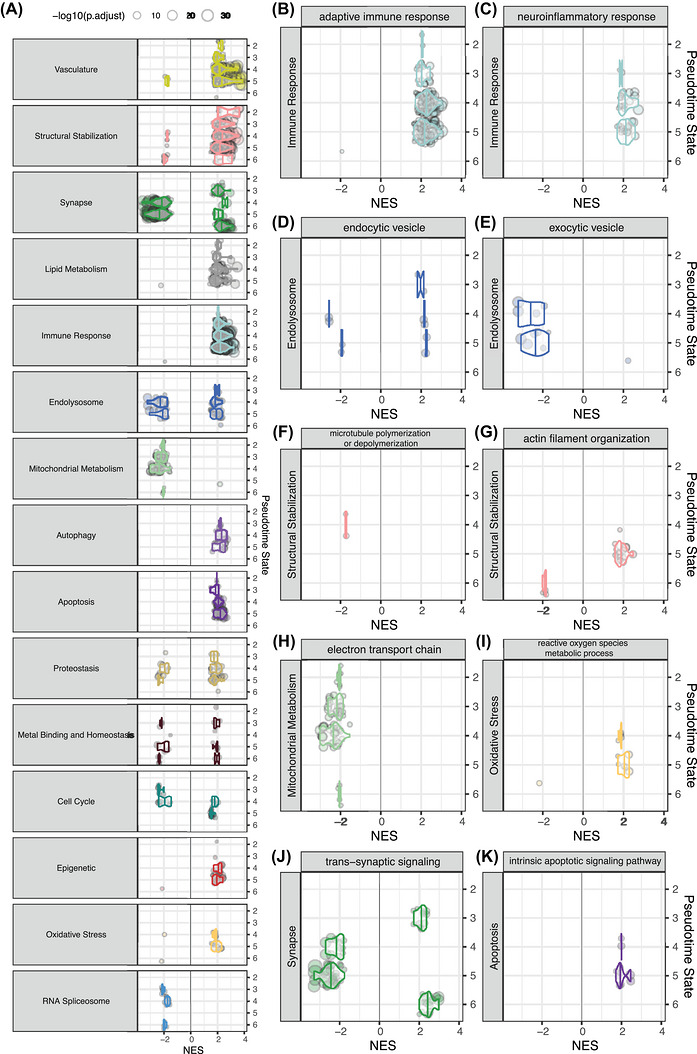
Transcriptomic pseudotemporal profiles by biodomain and select subdomains. Gene set enrichment analysis (GSEA) statistics from analyses using pseudotemporal state‐based effects from the Mayo cohort are shown for each biodomain (A) and select subdomains (B–K). Each point is a Gene Ontology (GO) term from the indicated biodomain (A) or subdomain (B–K), while the position along the *x*‐axis corresponds to the normalized enrichment score (NES) and the size of the point is scaled by the adjusted *p* value from GSEA, −log_10_ transformed. The analysis is performed for each state from the pseudotime trajectory, shown on the *y*‐axis, relative to the state with the largest proportion of control donors (i.e., state 1); state 2 represents relatively “early” events, while states 5 and 6 represent events later in disease pathogression. Term NES values indicate whether transcripts from the term tend to be upregulated (positive NES) or downregulated (negative NES) in *post mortem* Alzheimer's disease brains from donors in that pseudostate relative to state 1.

Cross‐sectional *post mortem* analyses are hampered by the lack of longitudinal information about AD progression. To address this, pseudotemporal modeling of *post mortem* samples has been employed to reconstruct pseudolongitudinal trajectories based on the molecular signature similarities between case and control brain donors from AMP‐AD cohorts.^2^ The pseudotime states are ordered relative to state 1, which was manually annotated because it contained the largest proportion of control donors, and the pseudotime state effect on gene expression was assessed relative to state 1. We used these pseudotime state effects to perform GSEA enrichment of GO terms and mapped the resulting terms onto biodomains and subdomains (Figures 5 and S7, Table S7).

In the original study, enrichments based on transcriptomic data from the Mayo cohort, which focused on temporal lobe expression, were relatively stronger than the enrichments from the ROSMAP cohort, which analyzed data from the dorsolateral prefrontal cortex. We note enrichment patterns similar to what was described in the previous work. By mapping results to the biodomains we could see that upregulation occurred early within the Immune Response, Structural Stabilization, Vasculature, Lipid Metabolism, and Apoptosis biodomains. Downregulation within Mitochondrial Metabolism begins at the earliest pseudotime states, and we noted bifurcated responses in the Synapse and Endolysosome biodomains.

Utilization of the subdomains refines these observations. For example, we noted that the *adaptive immune response* subdomain was upregulated early, beginning at pseudotime state 2 (Figure 5B), while the earliest upregulation of the *neuroinflammatory response* subdomain occurs slightly later at pseudotime state 3 (Figure 5C). While the Endolysosome domain is bifurcated, with some terms upregulated and some downregulated across pseudotime states, we noted an early upregulation of the *endocytic vesicle* subdomain (Figure 5D) at state 2 with later bifurcation of the subdomain terms. The *exocytic vesicle* subdomain terms, on the other hand, are nearly exclusively downregulated at states 4 and 5 (Figure 5E). Within the Structural Stabilization domain, we observed relatively earlier (state 4) downregulation of the *microtubule polymerization or depolymerization* subdomain (Figure 5F), with a later stage upregulation and subsequent downregulation of *actin filament organization* (Figure 5G). The pseudotemporally early downregulation within the Mitochondrial Metabolism biodomain appears mostly constrained to the *electron transport chain* subdomain, showing early downregulation beginning at state 2 and becoming more pronounced at later stages (Figure 5H). This precedes the upregulation of genes from the *reactive oxygen species metabolic process* subdomain (Figure 5I). We also noted flipped enrichment of terms from the *trans‐synaptic signaling* subdomain that are upregulated at states 3 and 6 and downregulated at states 4 and 5 (Figure 5J). The downregulation in *trans‐synaptic signaling* genes is coincident with the upregulation of genes from the *intrinsic apoptotic signaling* pathway (Figure 5K). Importantly, we see many similar patterns of enrichment, though moderately weaker, within the pseudotime analysis of ROSMAP transcriptomic data (Figure S7). These findings illustrate how mapping pseudotemporal trajectories onto the subdomain framework increases analytic granularity, uncovering dynamic shifts in specific biological subprocesses that remain obscured at broader domain scales. This approach provides a scalable model for integrating temporal context into multiomic analyses of neurodegeneration.

### 3.6 Subdomain characterization of gene perturbation datasets

CRISPRbrain is a public repository housing large‐scale functional genomics screens from iPSC‐derived, brain‐relevant cell types.^11^ Among the available resources are several CROP‐seq datasets, which combine pooled CRISPR perturbations with single‐cell RNA sequencing to profile transcriptomic response to gene perturbation at single‐cell resolution. CROP‐seq screens were performed in iPSC‐derived cell types from healthy donor iPSC lines, not patient‐derived iPSCs. The CRISPR perturbations are either activating (CRISPRa), where the expression of the target is increased, or inhibitory (CRISPRi), where the expression of the target is decreased. For this analysis, we utilized CROP‐seq data from CRISPR perturbations in iPSC‐derived glutamatergic neurons (CRISPRi *N* = 209, CRISPRa *N* = 97) and microglia (CRISPRi *N* = 39) – two cell types highly relevant to AD pathobiology. Although we did not generate new perturbation experiments for this study, these publicly available CRISPR perturbation datasets provide an orthogonal functional context for evaluating whether subdomain‐level gene sets yield interpretable and directionally consistent transcriptional responses.

**FIGURE 6:**
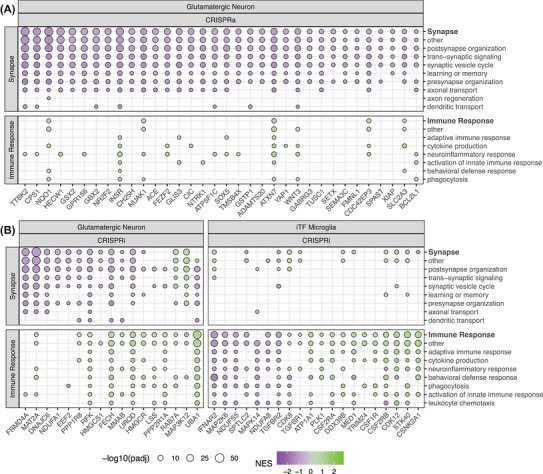
Transcriptomic effects of target perturbation in induced pluripotent stem cell (iPSC)‐derived neurons and microglia. Gene set enrichment analysis was performed against all genes annotated to each Alzheimer's disease biodomain and subdomain using transcriptomic results from CRISPRbrain CROP‐seq datasets in iPSC‐derived neurons and microglia. Results from the Synapse and Immune Response biodomains are shown (*y*‐axis) for targets (*x*‐axis) either activated via Clustered Regularly Interspaced Short Palindromic Repeats activating (CRISPRa) (A) or inhibited via CRISPR inhibitory (CRISPRi) (B) in each cell type. The size of each point corresponds to the adjusted *p* value, −log_10_ transformed, and the fill shows the NES for that domain or subdomain indicating whether the transcripts are generally up‐ or downregulated following perturbation of the indicated target. The parent biodomain is indicated in boldface. Results are shown only for targets that have significant enrichment for at least five subdomains across the two biodomains.

We used differential expression results from these perturbations to perform GSEA using genes annotated to each AD subdomain (Table S8). We focused on Synapse and Immune Response subdomains due to their cell‐type relevance. Overall, there were significant (FDR ≤ 0.05) enrichments for 49 (50.5%) of the CRISPRa perturbations in neurons, 52 (24.9%) of the CRISPRi perturbations in neurons, and 32 (82%) of the CRISPRi perturbations in microglia (Figure 6). These findings reveal broad perturbation‐induced transcriptomic changes and establish a basis for exploring how these effects impact functional domains in different cell types.

There was clear cell‐type specificity to the enrichment profiles. Perturbations in neurons predominantly enriched Synapse subdomains, whereas perturbations in microglia primarily enriched Immune Response subdomains. For targets showing enrichments in both domains, the direction of enrichment often diverged: when Immune Response genes were upregulated, Synapse genes tended to be downregulated, and vice versa. Across neuronal perturbations – regardless of CRISPR modality – the majority produced downregulation (i.e., negative NES) of Synapse domain genes. Only three targets (MAP3K12, RAB7A, and UBA1) showed any upregulated Synapse subdomains. In contrast, microglial perturbations exhibited more mixed outcomes, with roughly equal representation of targets that yield up‐ and downregulation in Immune Response subdomains (seven and eleven targets, respectively). These results reveal distinctions between how neuronal and microglial cells respond to genetic perturbation, with neurons showing widespread repression of Synapse domain genes, and microglia displaying a more heterogeneous response among Immune Response genes.

The subdomains yielded enhanced sensitivity, revealing processes affected by perturbation that were either not evident at the parent domain level or directionally distinct from other subdomains. Targets such as LSS, PPP2R1A, UBA1, and MAPK14 exhibited significant enrichment for individual subdomains, even in the absence of overall parent domain‐level significance. Furthermore, certain targets had subdomain‐specific directionality. For example, the RAB7A CRISPRi in neurons yielded upregulation across both domains overall, yet the *presynapse organization* subdomain was uniquely downregulated. Similarly, inhibition of CSF2RA or CSF2RB upregulated many Immune Response subdomains, but *leukocyte chemotaxis* was selectively downregulated when either of these targets was inhibited. These examples demonstrate how subdomain mapping detects subtle, process‐specific effects that are obscured at the parent domain level.

Given that AD pathology typically induces upregulation of Immune Response domain terms and downregulation of Synapse domain terms (e.g., Figures 4 and 6), we can use these data to identify targets that reverse these trends. In neurons, inhibition of RAB7A or MAP3K12 led to broad upregulation of Synapse subdomains, and for MAP3K12 there was also downregulation in several Immune Response subdomains. Furthermore, seven targets (IFNAR2, MAP2K6, NDUFS5, SPTLC2, MAPK14, NDUFA8, and TGFBR2) produced widespread downregulation of Immune Response subdomains. Notably, three of these (IFNAR2, MAPK14, and TGFBR2) also increased expression of genes from Synapse subdomains, particularly those annotated to *postsynapse organization*. Overall, these findings indicate that targeted perturbations can reverse disease‐associated transcriptomic signatures, promoting partial restoration of synaptic and immune homeostasis in AD‐relevant cell types.

## 4 DISCUSSION

This study refines the AD biodomain framework into 110 disease‐informed subdomains, increasing biological resolution while reducing redundancy across multiple data modalities. The primary goal of developing the AD subdomains was to create a framework that more precisely mapped disease‐implicated biological processes, enabling mechanistic hypothesis formation and therapeutic target identification. We described a novel methodology that integrates disease‐informed ranked gene sets (using the TRS^1^) with  kappa‐network modeling of shared gene annotation between GO terms. By defining connections between terms as edge weights proportional to gene conservation, we can identify connected subnetworks that represent coherent subprocesses of AD‐associated biology. We employed two complementary strategies to identify subdomains (Figure 1): a term‐centric (top‐down) approach, which constrains analyses within the biodomain structure, and a gene‐centric (bottom‐up) approach, which detects emergent clusters from strongly disease associated genes without prior domain constraint. Each offers distinct advantages – stability and interpretability for the former, sensitivity and discovery potential for the latter – and both converged on a consistent set of enriched biological processes (Figure S2). Harmonization of subdomains identified via these two approaches reduces methodological bias and reinforces data‐driven reproducibility.

The resulting 110 subdomains derived from the 19 parent biological domains exhibit limited GO‐term overlap but higher gene overlap (Figures 2,S3, and S4), mirroring the structure of the original biological domains.^1^ Overlapping GO terms and gene annotations between subdomains occur primarily at conceptual intersections – such as mitophagy between the Autophagy and Mitochondrial Metabolism domains – supporting the hierarchical yet interconnected architecture of AD biology (Figure 2B,C). A common challenge in evaluating ontology‐derived frameworks is the absence of a definitive ground truth against which biological subprocess boundaries can be benchmarked. Accordingly, validation of the subdomain framework was approached through robustness and reproducibility rather than direct benchmarking. The concordance between term‐driven and gene‐centric network construction strategies, the recurrence of core subdomains across independent datasets, and the internal coherence of gene annotations within subdomains support the stability of the framework. Collectively, these findings suggest that the identified subdomains represent robust and reproducible organizational features of the data.

Application of the subdomains to large‐scale AD datasets refined the precision of conclusions from those analyses. For example, the Mitochondrial Metabolism biodomain is one of the strongest enrichments from our integrated analyses of AD‐associated molecular signatures, and the subdomain enrichments localized this more precisely to the *electron transport chain* and *proton motive force‐driven ATP synthesis* subdomains (Figure 3). The *electron transport chain* subdomain enrichments are relatively stronger from the multiomics dimension, though they are still implicated by genetic risk (Figure S6), and enrichments from AMP‐AD proteomics data indicate that this subdomain is one of the most strongly downregulated in AD brains (Figure 4). We also noted that the *electron transport chain* subdomain is one of the earliest signals of downregulation in pseudotemporal models based on transcriptomic data from the temporal cortex and precedes the upregulation of the *reactive oxygen species metabolic process* subdomain (Figure 5). This convergence across data modalities highlights the mitochondrial electron transport chain as an early and cross‐modality vulnerability in AD. Consistent with mitochondrial hypometabolism being an early feature of disease pathogenesis,^13^, ^14^ as decreases in radiolabeled glucose uptake are common to subjective cognitive impairment,^15^ late‐onset AD^16^, ^17^ and familial AD,^18^, ^19^ these findings reinforce the translational rationale for targeting mitochondrial complex I and related bioenergetic pathways.^20^, ^21^, ^22^

Upregulation of Immune Response biodomain terms has been a robust and consistent signature of disease association. Using the subdomain framework, we refined this signal and highlighted the relative strength of the upregulation of the *adaptive immune response* subdomain relative to the *activation of innate immune response* subdomain (Figure 4) and noted that the upregulation of *adaptive immune response* subdomain transcripts was one of the earliest signals in the disease pseudotime from the temporal cortex (Figure 5). The emergence of adaptive immune pathways from this analysis is relatively surprising, given the broad focus on contributions of microglia to AD pathophysiology. While the *adaptive immune response* subdomain has a higher maximum NES value for TRS‐based enrichments, *activation of innate immune response* has a higher median NES value, which reinforces the importance of innate immune functions as contributors to AD. Moreover, when we investigated which specific terms were driving the strong enrichment within the *adaptive immune response* subdomain, we noted that the term with the strongest enrichment from the subdomain based on the TRS was “T cell activation via T cell receptor contact with antigen bound to MHC molecule on antigen presenting cell” (adjusted *p* value 1.8 × 10^−5^, NES 2.4), which sits at the interface of adaptive and innate immune arms. The prominence of the *adaptive immune response* subdomain reinforces emerging evidence that adaptive immunity plays a substantive role in AD pathogenesis,^23^, ^24^, ^25^ and that crosstalk between central nervous system (CNS) and peripheral immunity could provide a potent therapeutic entry point distinct from microglial modulation.

We noted several subdomains that showed opposing directions of enrichment between transcriptomic and proteomic datasets (Figure S6). Two of the seven terms significantly enriched from the *fatty acid metabolic process* subdomain are significantly upregulated in transcriptomic data but significantly downregulated in proteomic data. Among the genes driving enrichment for each modality we identified FDFT1, the first committed enzyme in the cholesterol biosynthesis pathway. Cholesterol metabolism has long been linked to AD pathogenesis, and the *cholesterol metabolic process* subdomain is the most strongly enriched of the Lipid Metabolism subdomains (Figure 3). FDFT1 itself ranks within the top 0.68% of all TRSs, including the top 3% for genetic scores and the top 4% for multiomic scores. A similar pattern was observed for the *receptor‐mediated endocytosis* subdomain, in which terms were upregulated in the transcriptome but downregulated in proteome‐based enrichments. A key gene driving enrichment for both modalities was PICALM, which is identified as a causal gene by the Alzheimer's Disease Sequencing Project's Gene Verification Committee (adsp.niagads.org), reported in ADGC GWAS meta‐analysis,^26^ and is among the top 1.2% of all TRS with equal contributions from genetic and multiomic scores (top 5.5% for each). These cross‐modality discrepancies likely represent points of molecular disequilibrium, where transcriptional output reflects attempts to compensate for deficits in protein abundance or function. While PICALM's role in lipid droplet formation is known,^27^ the role of FDFT1 is not explored in AD, suggesting an exciting therapeutic entry point implicated by this data‐driven approach.

The CRISPRbrain CROP‐seq resource provides an orthogonal perturbation‐based dataset for evaluating the functional interpretability of the subdomain framework. These publicly available CRISPR‐based transcriptional profiles enable assessment of whether subdomain gene sets yield coherent and directionally sensitive responses to targeted gene modulation in disease‐relevant cell types. The results revealed striking cell‐type specificity: Perturbations in neurons affect Synapse subdomains while perturbations in microglia primarily affected Immune Response subdomains (Figure 5). Subdomain enrichments captured nuanced effects that were missing or opposite at the parent domain level. Inhibition of UBA1 in neurons did not have a significant enrichment of the Synapse biodomain overall, but six of the seven Synapse subdomains were significantly enriched. Similarly, microglial inhibition of IFNAR2 led to broad downregulation of the Immune Response biodomain and most subdomains, but the *leukocyte chemotaxis* subdomain was upregulated. These examples highlight the sensitivity of the subdomain framework in isolating focal, mechanistically meaningful responses. Finally, we identified several perturbations within the dataset that induce reversals in AD‐associated patterns of dysregulation detailed above. Neuronal inhibition of RAB7A or MAP3K12 increased Synapse subdomain expression while microglial inhibition of IFNAR2, MAPK14, and TGFBR2 reduced the expression in many Immune Response subdomains. Such reversals provide functional context supporting the biological relevance and directional sensitivity of subdomain‐level enrichment and suggest that subdomain signatures may serve as actionable molecular readouts for target prioritization in a cell‐type‐specific manner.

There are several limitations to the current approach. As with the original biological domains, here we are also constrained by the accuracy and recency of the functional annotations in the GO. Furthermore, strict Cohen's  κ‐thresholds may have excluded weaker but biologically relevant associations from the term networks. Future work will test graded filtration approaches and integrate alternative network metrics to recover subtler, context‐dependent modules. Our current implementation also focuses on CNS biology, emphasizing processes most directly implicated in AD. Future development will focus on extending this work to peripheral systems, cellular development, and cross‐organ interactions (e.g., gut–brain axis). A further limitation is that we did not perform new experimental perturbation studies to validate specific candidate genes or pathways. Future work will focus on prospective validation of high‐priority subdomain‐linked targets using perturbation assays. Additionally, future work will characterize the relevancy of biodomains and subdomains to specific cell types.

## 5 CONCLUSIONS

Overall, this work demonstrates that large‐scale AD datasets can be projected onto disease‐informed subprocesses to gain interpretive precision and biological coherence. The subdomains enhance sensitivity for detecting specific molecular signatures, allowing more direct alignment of hypotheses, therapeutic interventions, and functional readouts. Beyond annotation, the subdomains provide a molecular fingerprinting framework to evaluate the ways in which disease models recapitulate AD biology, quantify the extent to which experimental perturbations reverse pathological signatures, and ultimately uncover novel therapeutic avenues grounded in a mechanistic context.

## CONFLICT OF INTEREST STATEMENT

Gregory A. Cary, Sai Sruthi Amirtha Ganesh, Laura Heath, Karina Leal, Robert R. Butler III, and Jesse Wiley declare no conflicts of interest. Martin Kampmann is a co‐scientific founder of Montara Therapeutics, serves on the scientific advisory boards of Montara Therapeutics, Engine Biosciences, Alector, and Neurocrine, and is an advisor to Modulo Bio and Theseus Therapies. Martin Kampmann is an inventor on US Patent 11,254,933 related to CRISPRi and CRISPRa screening, and on a US patent application on in vivo screening methods. Frank M. Longo is a board member, equity owner, and paid consultant for PharmatrophiX, a company focused on the development of small molecule ligands for neurotrophin receptors. Allan L. Levey is a paid consultant for EmTheraPro, Cognito Therapeutics, Cognition Therapeutics, and Alamar. Gregory W. Carter is a paid consultant for Astrex Pharmaceuticals. Author disclosures are available in the Supporting Information.

## CONSENT STATEMENT

No human participants were recruited for this work. The details of the Institutional Review Board (IRB)/oversight body that provided approval or exemption for the research described are given as follows: Western IRB–Copernicus Group (WCG) IRB of Sage Bionetworks gave ethical approval for this work.

## Supporting information



Supporting Information

Supporting Information

Supporting Information
