# Supplementary material for: Alzheimer's disease biological domain sub‐stratification enhances the precision of functional analyses
Source: Alzheimers Dement. 2026 Jun 10;22(6):e71403. doi: 10.1002/alz.71403 (PMC13253357; doi:10.1002/alz.71403)
Supplement: Supplementary file 3 — Supporting Information [file ALZ-22-e71403-s002.pdf]

Alzheimer's Disease Biological Domain Sub-Stratification Enhances the Precision of Functional Analyses

Supplementary Figures

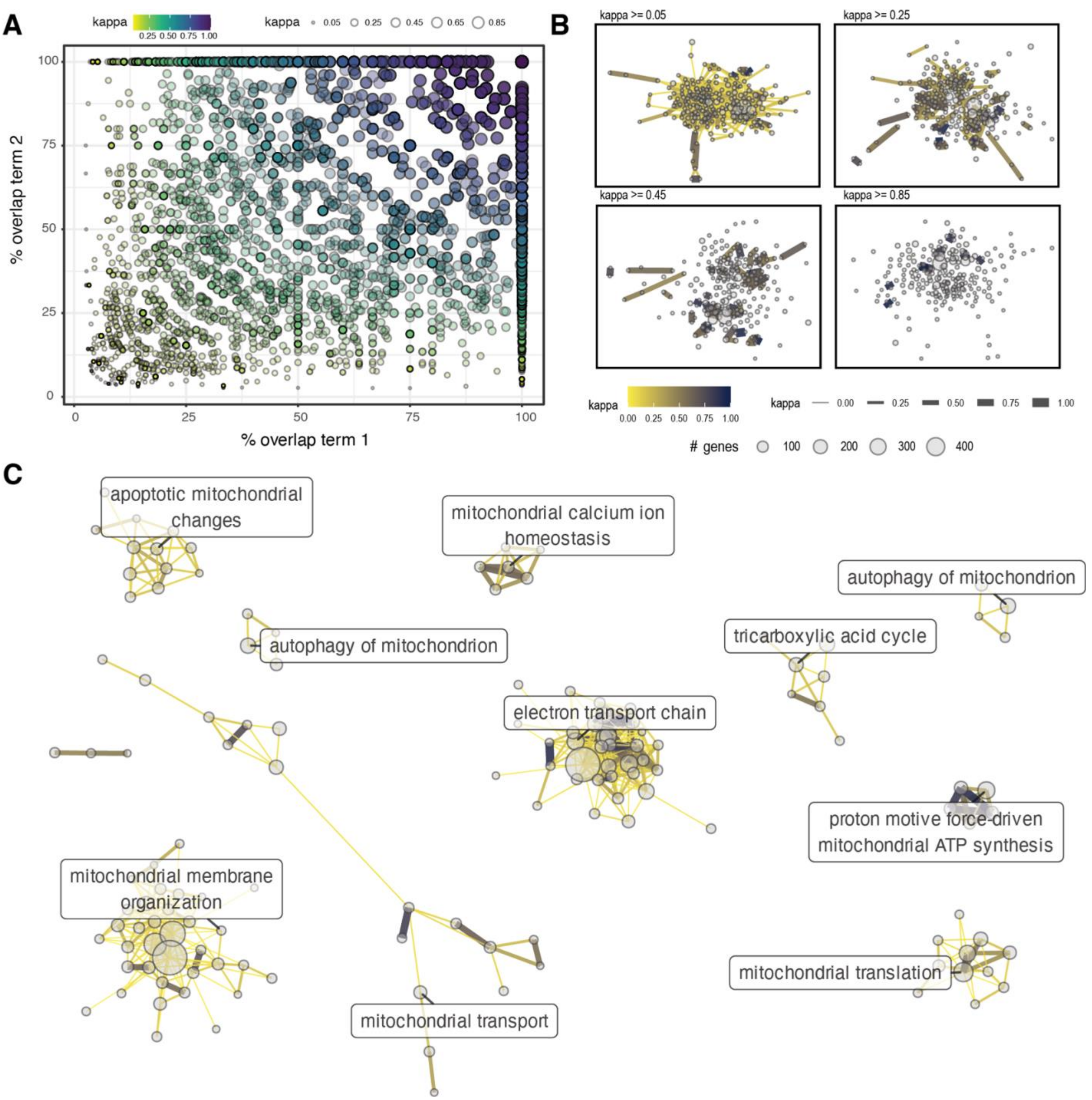

Supplementary Figure 1. Example of κ-network fragmentation and subdomain definition. (A) Subset of GO term pairs showing the percent of genes overlapping each term on the x-axis and y-axis and the computed Cohen's κ coefficient as the point fill. (B) Sensitivity analysis showing biodomain term network fragmentation at progressively increasing κ value thresholds. (C) Mitochondrial Metabolism biodomain terms fragmented into subdomains using the κ network filtering as described in text.

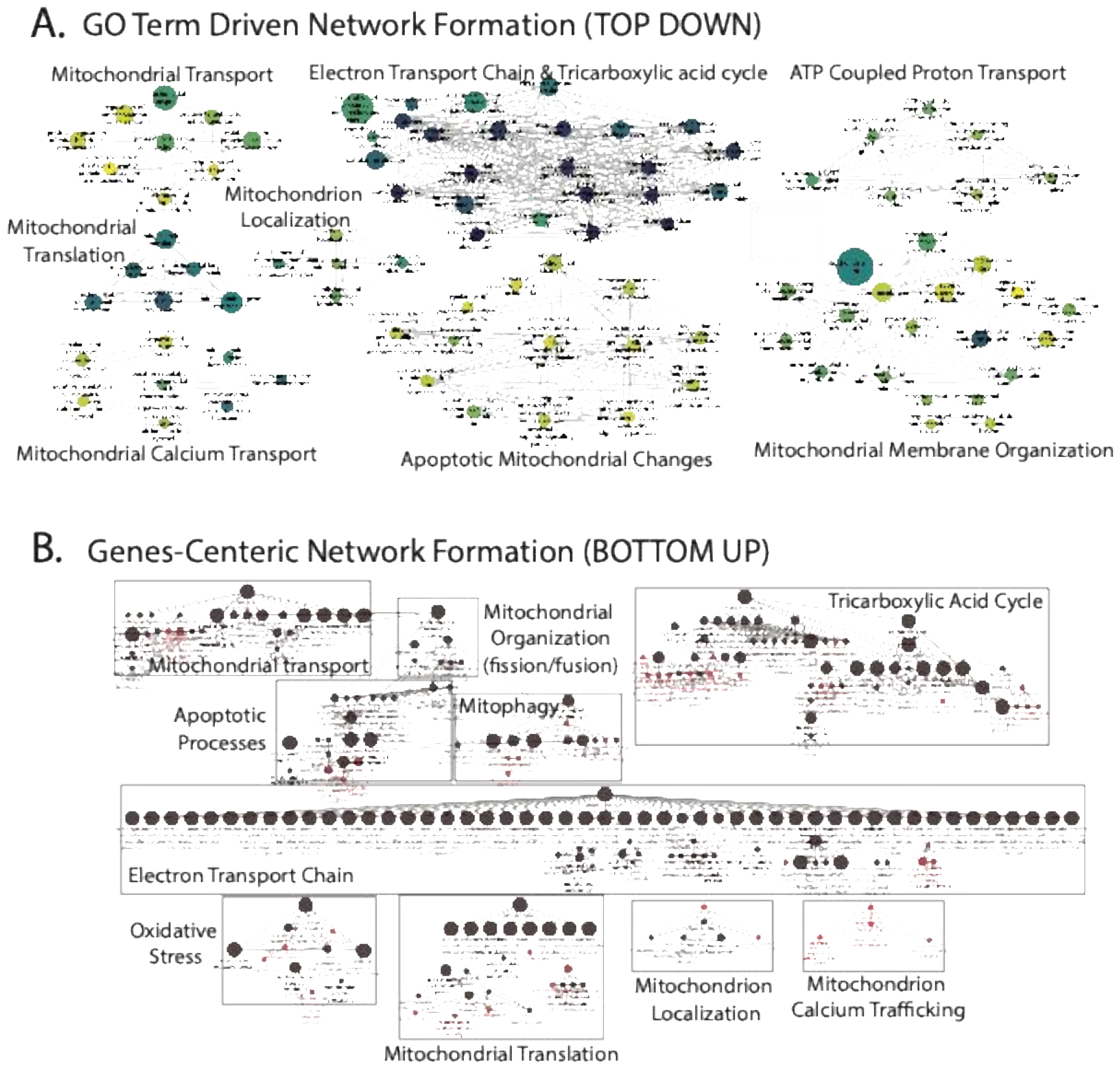

Supplementary Figure 2. Top-down and Bottom-up Modules from Mitochondrial Metabolism. κ-networks were developed for the mitochondrial metabolism biodomain from the set of GSEA enriched GO terms (A) and the GSEA leading edge genes (B). Each network from filtered for a κ-value of 0.5, yielding submodules when eliminating the weaker linking gene annotations from the network. Both approaches converge on a near identical set of submodules, suggesting that the top-down (A) and bottom-up (B) approach result in the identification of highly similar submodules.

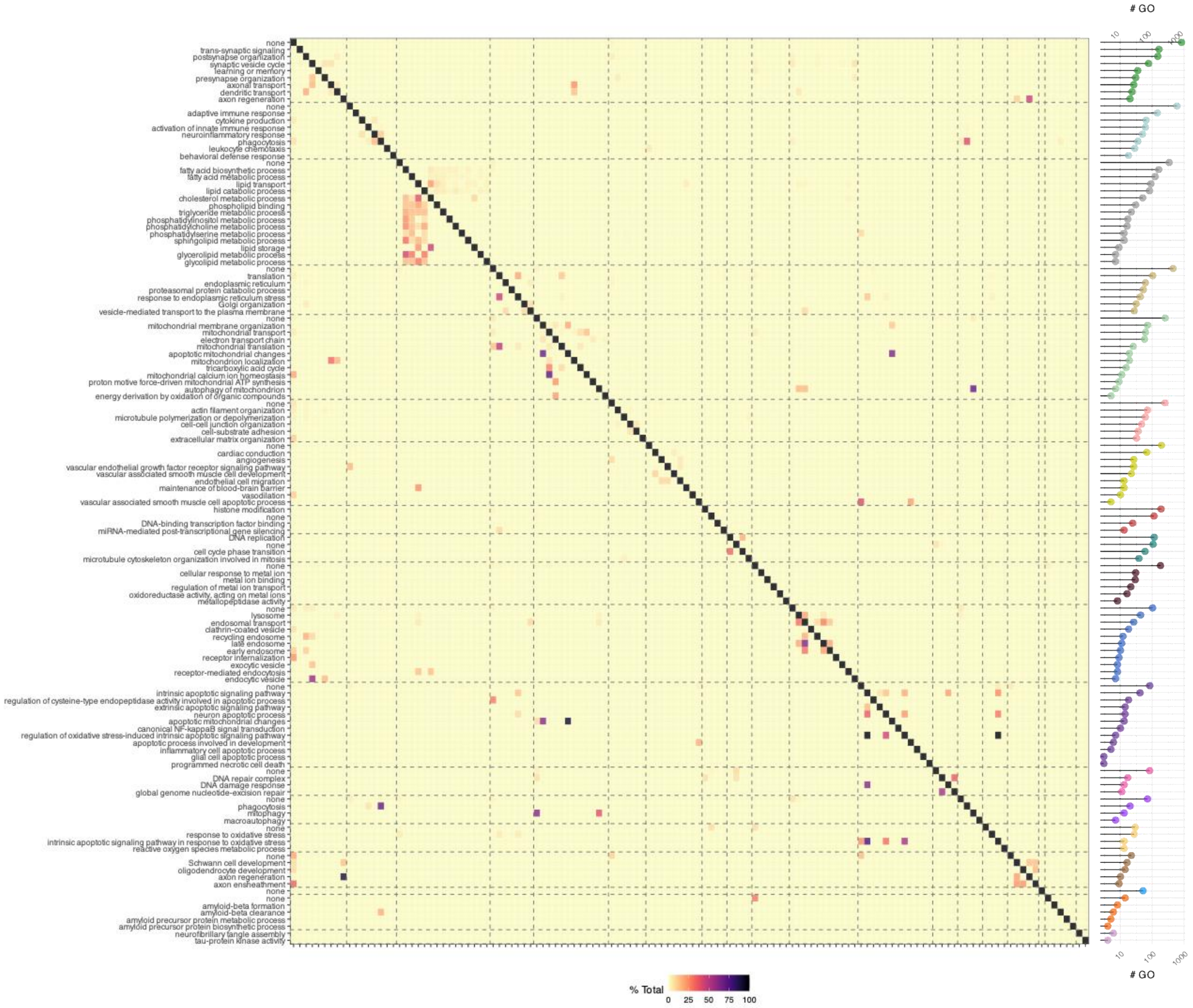

Supplementary Figure 3. GO term overlap across all subdomains. The subdomain label is shown along the y-axis and the number of GO terms within each subdomain is indicated by the lollipop plot on the right. The heatmap shows the percent of all annotated GO terms that overlap between subdomains. The ordering of subdomains is identical on the y- and x-axes.

Supplementary Figure 4

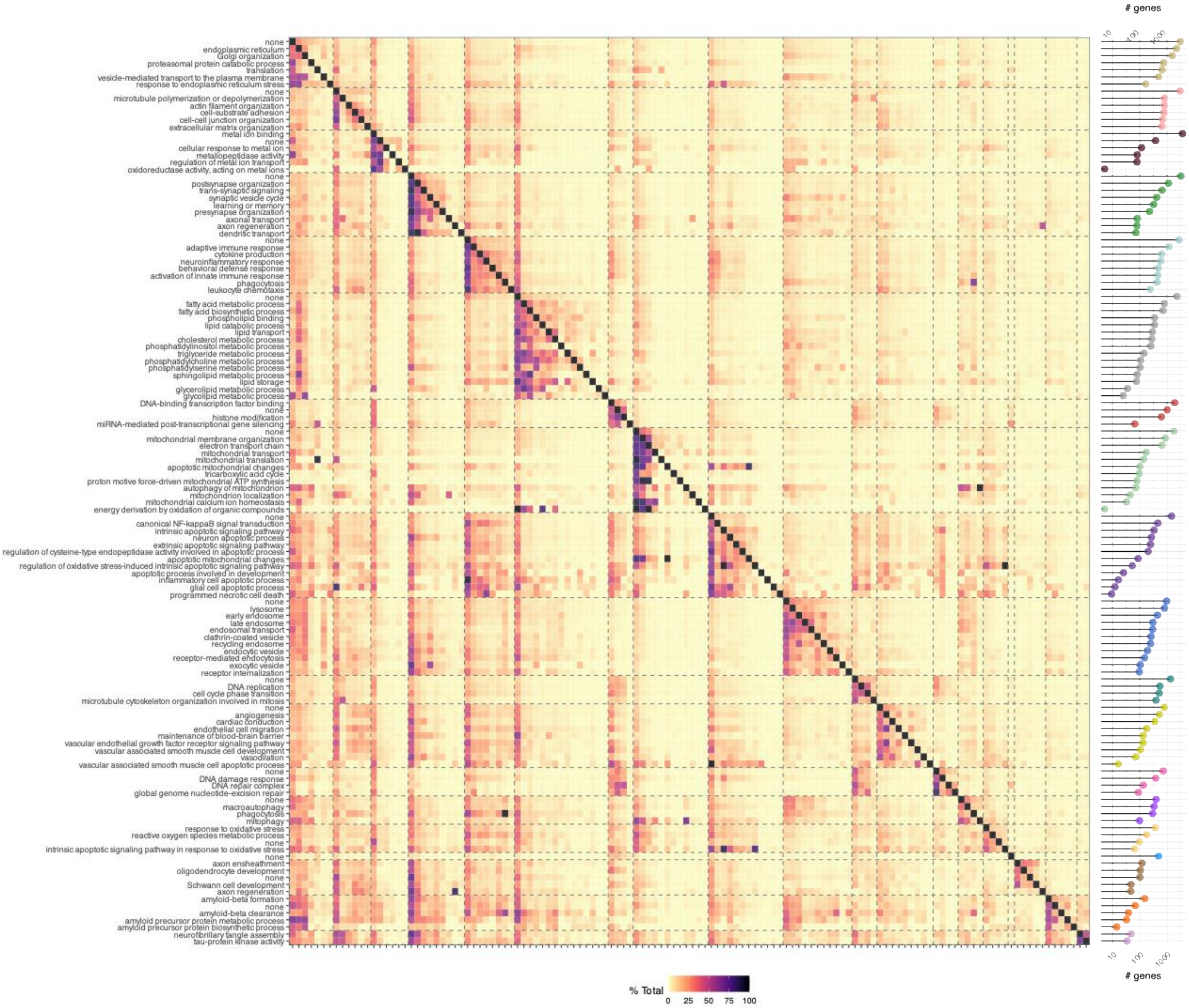

Supplementary Figure 4. Gene overlap across all subdomains. The subdomain label is shown along the y-axis and the number of genes annotated to each subdomain is indicated by the lollipop plot on the right. The heatmap shows the percent of all annotated genes that overlap between subdomains. The ordering of subdomains is identical on the y- and x-axes.

Supplementary Figure 5

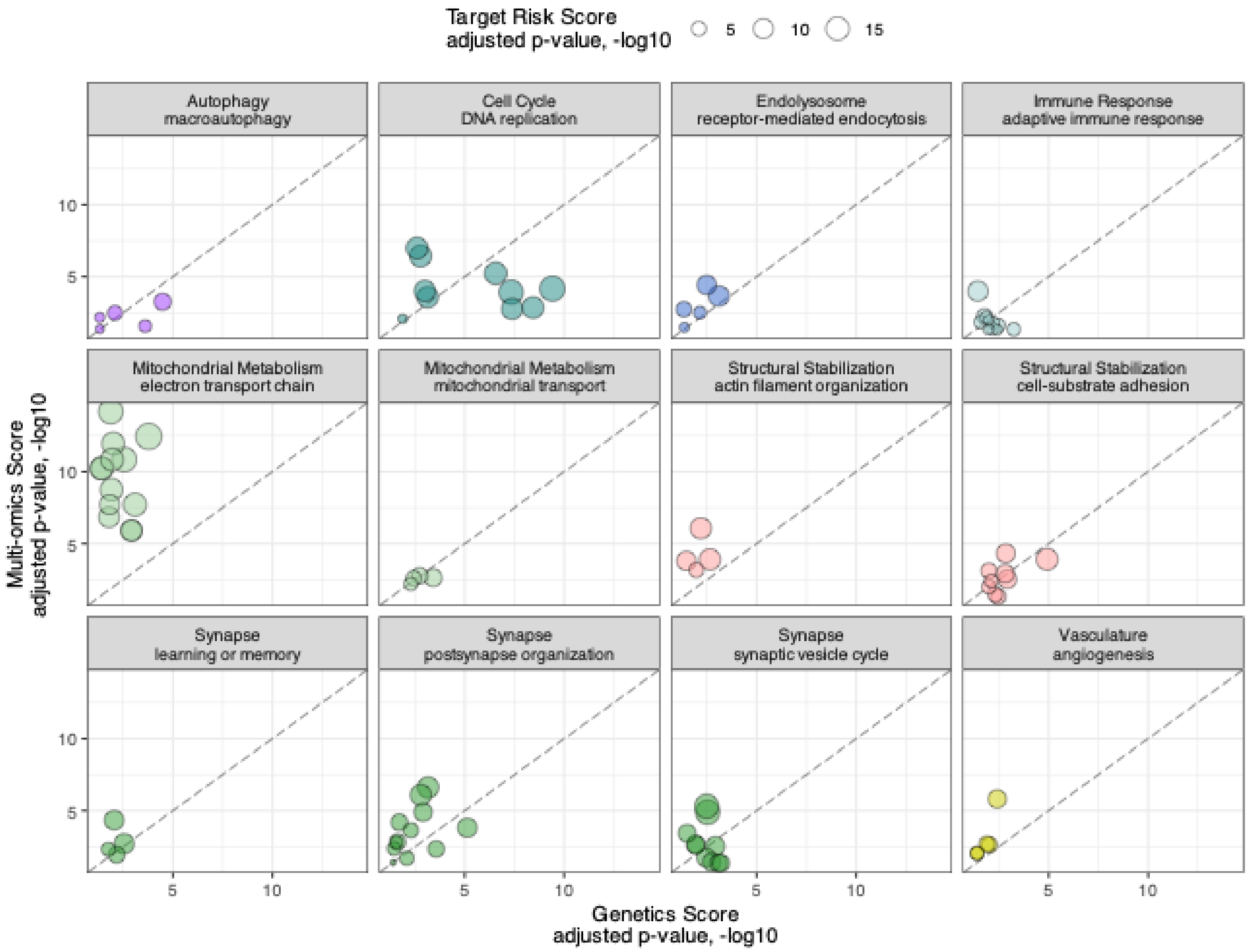

Supplementary Figure 5. Contrasting GSEA results between genetic and multi-omic component risk scores. For GO terms within each subdomain (facet) that are significantly enriched by both scores, the significance from the genetics score based enrichment is shown on the x-axis and the score from the multi-omic score enrichment is shown on the y-axis. In each plot the identity line is shown as a dashed line, and the size of each point corresponds to the significance of the enrichment for that term from the target risk score based GSEA.

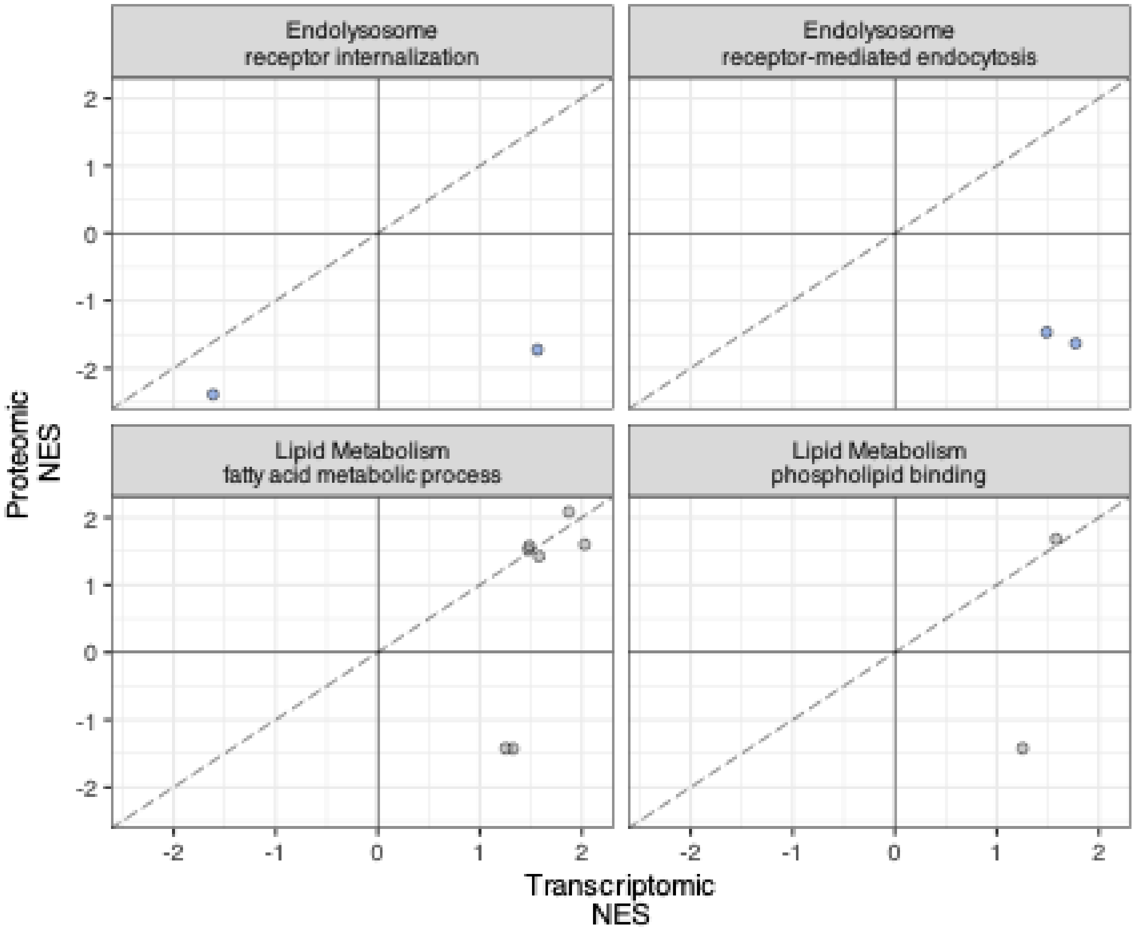

Supplementary Figure 6. Contrasting GSEA results between transcriptomic and proteomic meta-analyses. For GO terms within each subdomain (facet) that are significantly enriched by both datasets and with terms that are enriched with opposite signs, the NES from the transcriptomic based enrichment is shown on the x-axis and the NES from the proteomic based enrichment is shown on the y-axis. In each plot the identity line is shown as a dashed line.

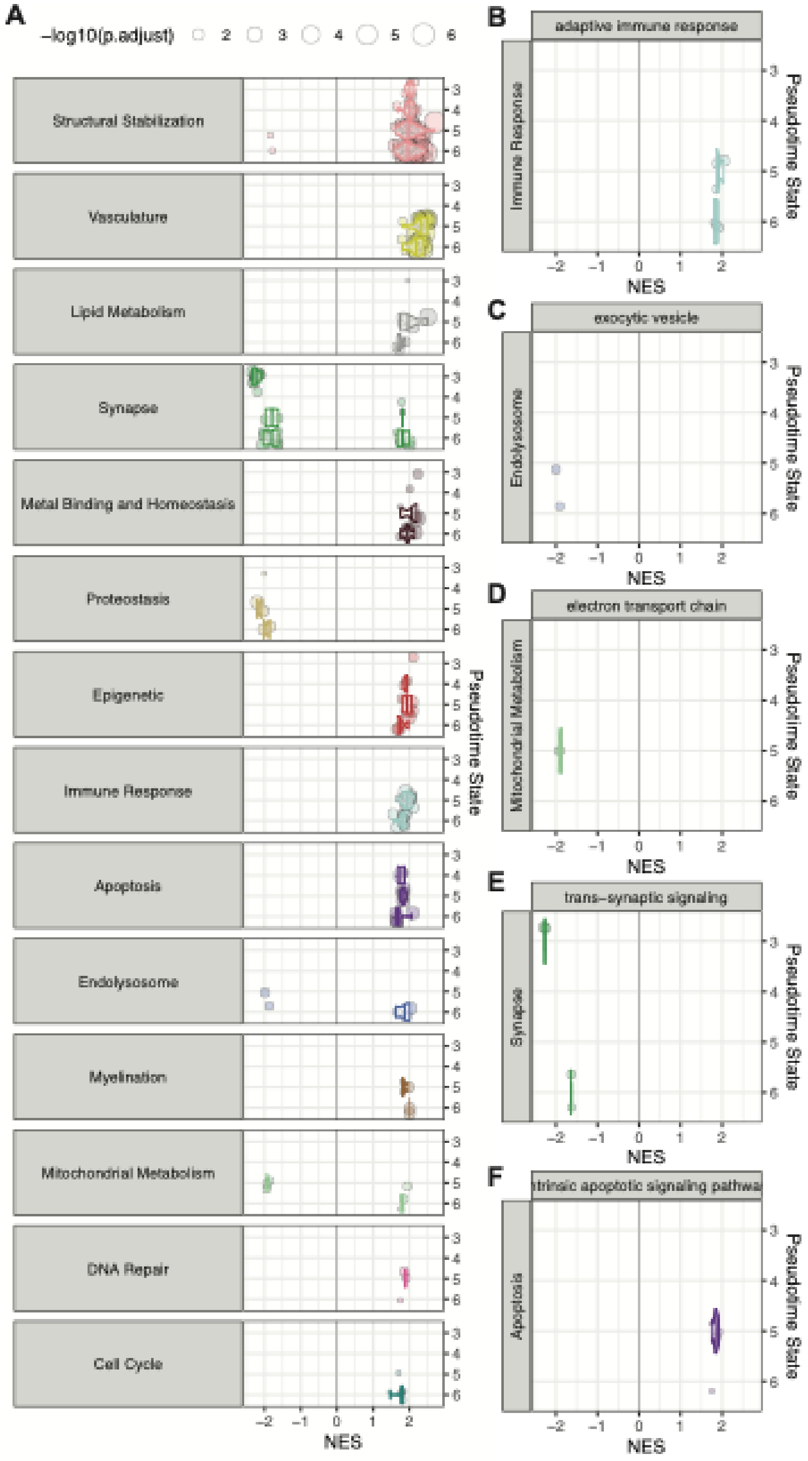

Supplementary Figure 7. GSEA statistics from analyses using pseudotemporal state-based effects from the ROSMAP cohort are shown for each biodomain (A) and select subdomains (B-F). Each point is a GO term from the indicated biodomain (A) or subdomain (B-F) while the position along the x-axis corresponds to the normalized enrichment score (NES) and the size of the point is scaled by the adjusted p-value from GSEA, -$\log_{10}$ transformed. The analysis is performed for each state from the pseudotime trajectory, shown on the y-axis, relative to the state with the largest proportion of control donors (i.e., state 1); state 3 represents relatively "early" events, while states 5 and 6 represent events later in disease pathogression. Term NES values indicate whether transcripts from the term tend to be up-regulated (positive NES) or down-regulated (negative NES) in postmortem AD brains from donors in that pseudo-state relative to state 1.
